# Contagious Peripneumonia and Preventive Inoculation

**Published:** 1882-03

**Authors:** 


### CONTAGIOUS PERIPNEUMONIA AND PREVENTIVE INOCULATION.

M. Leblanc declares himself opposed to inoculation and bases his experience upon a large number of observations. The following are his conclusions:

1. Contagious peripneumonia of cattle may develop itself spontaneously in certain countries, and from certain influences known for the last century.

2. The effects of inoculations present such variations as much in regard to evolution as to intensity and secondary accidents, that they cannot be regarded as analogous to those obtained in other diseases.

.3. Inoculation with pulmonary serum does provoke an analogous affection, in an attenuated form, and in case of death, none of the characteristic lesions of the disease are present.

4. Inoculation, in many cases, is powerless to confer immunity even for a short time.

5. The preservative power cannot be longer than six months as the experiments in re-inoculation tend to show.

6. The strict execution of the measure prescribed by sanitary regulations would give as satisfactory results and less costly.—*Paris Medical.*

